# Rapid progress or lengthy process? electronic personal health records in mental health

**DOI:** 10.1186/1471-244X-11-117

**Published:** 2011-07-26

**Authors:** Liam Ennis, Diana Rose, Felicity Callard, Mike Denis, Til Wykes

**Affiliations:** 1 Health Services and Population Research Department, Institute of Psychiatry, London SE5 8AF; UK; 2 ICT Department, South London and Maudsley NHS Foundation Trust, Newman Road, London BR1 1RJ; UK; 3 Department of Psychology, Institute of Psychiatry, London SE5 8AF; UK

## Abstract

A major objective of many healthcare providers is to increase patients' participation in their own care. The introduction of electronic personal health records (ePHRs) may help to achieve this. An ePHR is an electronic database of an individual's health information, accessible to and maintained by the patient. ePHRs are very much in vogue, with an increasing number of studies reporting their potential utility as well as cost. However, the vast majority of these studies focus on general healthcare. Little attempt has been made to document the specific problems which might occur throughout the implementation of ePHRs in mental health. This review identifies such concerns through an electronic search of the literature. Several potential difficulties are highlighted and addressed, including access to information technology, identifying relevant populations and the handling of sensitive information. Special attention is paid to the concept of 'empowerment' and what this means in relation to ePHRs.

## Background

The NHS Connecting for Health system is an attempt by the UK government to coordinate all levels of care (primary, secondary, tertiary and community) using electronic health records, begun in 2002. The programme has been unsuccessful and by the end of 2008 had failed to achieve savings or allay doubts of doctors or patients about its clinical utility or data security; additionally, the programme is hugely over budget [1]. However, there has been continued attention in the UK recently to the introduction of the internet into health care. There is a large amount of optimism that it will be possible to improve health using new technology with information and therapies being available directly to NHS patients. NICE now recommends computerised CBT for depression [2]. There have also been several recent grant announcements from the MRC, National Institute for Health Research (NIHR) and charities such as the Wellcome Trust for developments of new and remote internet technologies that will improve healthcare in the elderly and people with chronic conditions. There is optimism that these technologies will prove beneficial across all health conditions, but the adoption of these systems into mental health care may prove more difficult. This review investigates likely problems using current research on one aspect of these technologies, electronic personal health records (ePHRs).

The adoption of electronic personal health records - an electronic database of an individual's health information, accessible to and maintained by the patient - is an important goal for many healthcare providers [3,4]. The purported benefits are wide-ranging, including increased quality of care, reduction in medical errors and reduced costs [5,6], as well as providing an unprecedented rich source of data regarding population health. This is especially so given the recent development of search tools with the capability of pseudonymising and retrieving patient records such as the Case Register Interactive Search tool (CRIS)[7]. ePHRs are also likely, particularly in mental health care, to offer the opportunity of sharing information between services and with the individual service user so information does not get lost. This has been a problem as individuals using long term mental health care services often lose some continuity of care as they move from one service to another depending on their current difficulties [8,9]. ePHRs are favoured by the UK government whose aim is to increase empowerment and allow the patient more engagement in their treatment options [3]. The US government is similarly enthusiastic and has invested in a $19 billion programme to improve health information technology and electronic health records in particular [4].

Much of the information on electronic records comes from general medical care and therefore one cannot assume that it will relate to people who have mental health difficulties. Electronic information storage and exchange is surrounded by sensitivities and potential for stigma and discrimination in regard to those with psychiatric diagnoses [10]. However, these initial attempts to embed personal electronic health systems can indicate the challenges and design solutions for those in touch with mental health services.

### What we can learn from general medical systems

Certain systems are already in use with very encouraging results. Kaiser Permanente, a large healthcare organisation based in the US, began initiating their ePHR in 2004. The most recently published figures showed that registration had been rising steadily, and by June 2008, 27% of its 8.7 million patients had registered on their website using a variety of online facilities such as obtaining test results, refilling prescriptions and emailing doctors [11]. The frequency of use of these online facilities has been increasing rapidly, with just over 62% of registered patients using them twice or more over a 6 month period, presumably demonstrating that individuals find the services useful once they have engaged with them [11]. Other research on the Kaiser Permanente model showed that the online services resulted in a significant decrease in the total number of office visits per patient from 2004 to 2007 [12]. Despite this success, other systems have not met with the same enthusiasm. A British equivalent of the ePHR named HealthSpace was introduced across the NHS in 2007. The HealthSpace system consisted of two levels; a basic account, which is essentially a depository for an individual's health information which individuals must complete themselves, and an advanced account, through which they could access their summary care records, book appointments and communicate remotely with practitioners. At the time of the launch of the advanced HealthSpace account, there were high hopes for uptake of the system and its positive effects [13]. Three years later Greenhalgh, Hinder, Stramer, Bratan and Russell found that just 0.13% of those who possessed a basic account went on to open an advanced account when invited [14], as compared to the anticipated 5-10% uptake.

Greenhalgh et al.'s evaluation of HealthSpace [14,15] highlighted a number of important distinctions between the Kaiser Permanente model and HealthSpace. Firstly, the HealthSpace system was regarded as counter-intuitive, cumbersome and generally difficult to use. The system was equally deemed to be uninteresting, as the basic account only contained information which had been provided by the users themselves, and the registration process to open an advanced account was complicated and laborious. The key flaw with HealthSpace was that it was designed by the service provider rather than the service user. This resulted in a system containing large disparities between what the consumers were *thought *to want and what they actually wanted. Crucially, those patients with a chronic illness whom the researchers interviewed emphasised that 'self monitoring of health data involves a complex interaction between patient and clinician and that the process of entering and accessing data cannot be meaningfully separated from the wider care relationship'. Users therefore saw little gain in entering data that would be seen only by themselves, rather than data that could contribute to and build upon their clinical relationships. Such comments from patients with a chronic illness have potential implications for the introduction of ePHRs in mental health care, given the lengthy periods of contact that many service users have with mental health services, and the importance of the therapeutic relationship to healthcare outcomes. Thus the success of the Kaiser Permanente system, taken with the relative failure of HealthSpace, shows that expectations regarding technological transformations do not always translate into real gains.

### Attitudes towards ePHRs in mental health

The trumpeting of self-determination in health decisions has often not extended from the general population to the sphere of psychiatry [16]. This is rapidly changing, and as transparency also becomes a significant issue, psychiatrists can expect to be asked to defend their decisions to block patient access to records on the grounds that it will incur 'serious harm' [17]. These shifts in attitude represent important changes in the equality of mental health service users. As with any paradigm shift, there is a danger of new ideas being advanced in the absence of strong empirical support. At the very least, it is important to understand if mental health service users would want to use these technologies - and for what purposes - before embarking on the large-scale implementation of new ePHR systems. A Google Scholar search in March 2011 using the terms "ELECTRONIC PERSONAL HEALTH RECORD" returned 313 000 results; when the word "MENTAL" was added this dropped to 123 000 results. On closer inspection, most articles returned mentioned the word "MENTAL" only once, and in the overwhelming majority of cases it was used simply as an example of the type of information which warrants *exclusion *from an ePHR. The lesson is that if failures such as that of HealthSpace can occur in spite of a relatively large body of evidence, then we must be even more careful when the literature is sparse; the potential pitfalls of implementing ePHRs which are specific to the field of mental health are highlighted here.

### What criteria need to be fulfilled in order to implement an ePHR in mental health?

#### (i) Identifying relevant populations

The wide-spread adoption of an ePHR is only tenable if the target population have access to and are able to use the new mediums of communication. Borzekowski et al found that amongst their US sample of 100 outpatients experiencing severe mental illness, only 36 had ever used the internet, and just 28 owned their own computer [18]. Amongst the reported barriers to using the internet were cost, lack of access, cognitive difficulties, lack of computer literacy and typing problems. Although interest in using the internet was high amongst this sample, each of these barriers represents a substantial task for ePHR initiatives in the field of mental health. IT skills training may be necessary to enlist non-users of the internet, along with making access to the internet (in whichever form the ePHR adopts) readily available free of charge, especially among older and less-educated subpopulations. Other demographics, as well as age and education, must also be taken into account. Amongst both US and British samples there have been concerns that steps towards shared care in mental health may be more difficult amongst those with poor introspection, as those who deny the existence of their illness might find notes distressing [19,20]. In addition, Essex et al. [19] found that in a sample of people in the UK mental health services, the use and acceptability of paper-based patient held records was lowest amongst those who were experiencing high levels of paranoia and delusions regarding state or media conspiracies. These individuals were particularly concerned about the records falling into the wrong hands. At this stage it is unclear whether electronic records would increase or decrease these anxieties should PHRs be introduced.

#### (ii) Professional participation

Sharing information has been an often voiced maxim but the experience has not been without its problems. Although copying patients into correspondence between clinicians became policy in the UK some time ago [21], the practice is frequently not adhered to. In fact a recent audit found that only 59% of letters sent were copied to patients [22]. In addition Bhandari found that these letters frequently required a standard of literacy that cannot be assumed in the normal population [22]. The level of literacy among some people in the mental health population is likely to be low as many mental health problems start in adolescence and therefore interfere with educational achievements. Essex, Doig and Renshaw [19] found that clinical staff in their UK sample were generally very unenthusiastic about introducing a patient-held record, with none of the 25 psychiatrists who were approached accepting the invitation to participate in the study. The psychiatrists reported that they were reluctant to meet with GPs to discuss shared care and were unhappy about GPs attempting to participate in areas in which they had little expertise [19]. Plovnick [23] suggested that clinicians may also resist the implementation of ePHRs, as initially they are likely to slow productivity. In addition, he raises concerns that copy and paste operations available on electronic systems could lead to impersonal and inaccurate clinical notes [23]. An obvious problem to address therefore is to ensure that clinicians are contributing to ePHRs in ways that they consider useful and beneficial in relation to their own practices of clinical care.

The reason for the lack of adherence to DoH guidelines - including the frequent omission of important information - is often cited as a desire to protect the patient from distress [24]. Although it is clear that clinicians have a duty of care - especially to disclosure of third party information provided confidentially - there is probably an overemphasis on reducing the information available. Allowing patients to access their health records may be a distressing experience for some. Bernadt, Gunning and Quenstedt investigated British patients' opinions of their own psychiatric records, and found that 28% of patients were upset by what they had read, as compared to only 4% who were upset by what had been said during their clinical interview [25]. In a more recent UK study Tahir, Bisson and Wilcox [26] found that only 37% of clinical staff agreed that copying letters to patients was a good idea as compared to 83% of patients, with the main concerns being reported as damaging the therapeutic relationship and causing anxiety amongst patients due to misinterpretation of the notes. Nandhra et al. [24] found that copying UK patients into letters sent from their psychiatrists to their GPs caused initial distress in nearly one in every five patients. However, 78% of patients also reported that it was helpful to receive the letter, and 83% reported that they would like to continue receiving them. In fact, Lepping, Paravastu, Turner, Billings & Minchom found that UK psychiatric patients were significantly *more *likely to want access to letters concerning their health than other medical patients [27]. Taken together these studies suggest that patients value access to clinical information and would have an appetite for continuing with this process. However, there is a need to ensure that clinicians are informed of how best to write notes that are comprehensible to, as well as not stigmatizing of patients, whilst appreciating that clinicians have concerns about promoting an open therapeutic relationship, and simultaneously protecting the patient from significant harm.

#### (iii) Data security and issues of confidence

As with any electronic system dealing with personal information, concerns have been identified regarding the security of ePHRs, especially given the highly sensitive nature of psychiatric information. In the US Weitzman, Kaci and Mandl used a thematic analysis technique to establish implications for the design of an ePHR in a non-psychiatric setting, and found that there were moderate concerns regarding potential breaches of privacy should an ePHR be implemented [28]. It is worth noting that some of the narrative analysed in Weitzman, Kaci and Mandl's study specifically refers to breaches of privacy being of greater concern should the individual suffer from a sensitive problem [28]. Equally there were concerns raised by samples in the US about who would be able to access information stored in the ePHR, for example whether insurance providers or employers would be able to view the information and consequently discriminate against individuals with health problems [28]. Using a similar method with an older population in Australia, Forsyth, Maddock, Iedema and Lassere [29] also found that whilst their non-psychiatric sample were not particularly concerned about privacy issues, certain participants believed that they would feel differently if they suffered from a mental illness. Plovnick stated that implementing an ePHR, which is accessible to all clinical staff responsible for the care of an individual, will necessarily increase the number of people who can access the sensitive information [23]. As a result there is greater scope for records to become dispersed amongst those not authorised to view the information, as well as greater difficulty in establishing where the 'leak' in the information loop has occurred [23]. This is again of potential importance for those service users who experience symptoms, such as paranoia or media-related delusions, as it might exacerbate their difficulties.

Along with security and privacy concerns, there are several studies which suggest that uptake of ePHRs may be hampered by concerns regarding the locus of responsibility. Weitzman, Kaci and Mandl [28] found that the 'desire for inclusion' was a significant motivator in adopting a personal health record in the US, and therefore it must be made clear to users that their input will be considered in clinical decision making. However, in the same study it was also noted that uncertainty regarding responsibility for maintaining the record was a significant barrier to adoption [28]. Consequently the implementation of ePHRs must include clear identification of boundaries between the expected input (and associated effects of this input) of both clinicians and users. This view has been echoed in specific psychiatric settings in which it has been shown, using an analogue scale of preferred decision making, that psychiatric patients would like equal participation in their treatment decisions with clinicians [20,30]. This level of shared decision making must be agreed upon and operationalised in order to avoid confusion regarding issues of accountability. For all those in the care package to be confident, it must be clear that some third party information could not be revealed automatically. This would preserve carers' confidence that when providing sensitive information, that this information will not be revealed to the service user without prior consent. Likewise, service users would need to have confidence that they maintained control over dissemination of material discussed confidentially with a care provider.

#### (iv) Organisational and clinical context

Any introduction of new technology into healthcare settings needs to take into account existing organizational and clinical practices and frameworks. There is frequently the temptation to fall into a technological determinism: policy makers and implementers hope that certain inherent features of the technology will bring about anticipated effects, without considering how the technology is part of a socio-technical complex comprising diverse structures and actors. Technologies have the capacity to shape existing roles, identities and expectations between service users and healthcare professionals, as well as between different actors within the healthcare system [31]. But the specific ways in which they do so depends significantly on a dynamic process that shifts across time and works out differently in diverse contexts. Any introduction of ePHRs in mental health needs therefore to attend carefully to how different healthcare contexts are likely to offer different constraints and potentials. Both the design and the implementation of ePHRs need to be flexible enough to respond nimbly to these dynamic landscapes.

### Evidence of the benefits and costs of implementing ePHRs in mental health

Despite the number of potential problems specific to ePHRs in mental health - and the past failings of other ePHRs such as Health Space - very few controlled studies exist in the literature. This was noted in Henderson and Laugharne's review [32], which identified no randomised controlled trials concerning patient-held records in mental health, in spite of considerable contemporary approval. Despite patient-held records being in use for those with mental ill health, there was no acceptable evidence which could assess the benefits or harms. Since this review, only two RCTs have been published but neither of these studies used an electronic record. Warner, King, Blizard, McClenahan and Tang conducted a 12-month cluster-randomised controlled trial in the UK using a paper-based patient held record, and found that there were no significant effects in the experimental group on symptom intensity, satisfaction with services or hospital admissions [33]. Furthermore these authors found that the use of the shared care record was low amongst both professionals and patients. The second RCT found better uptake of the UK-trialled (and again paper-based) patient held record, but replicated Warner et al. [33] in finding no significant differences between groups on measures of symptoms, satisfaction or use of services [34].

The lack of positive (or detrimental) effects may be due to a number of reasons. Both studies may have missed important effects due to the high attrition rates [35]. For example, in Warner et al.'s study 56% of the 90 participants randomised to PHR did not use it at all and were therefore deemed not to benefit from it [33]. Second, the outcome measures might not have been appropriate to identify the costs and benefits. Essex, Doig and Renshaw [19] showed that patient-held shared care records were acceptable to those with severe mental illness and improved autonomy, effectiveness of shared care, and communication with clinicians; none of these outcomes were assessed in either of the RCTs. In their review of patient held records in mental health services, Laugharne and Stafford [36] also discovered that patients generally found their notes useful, informative and felt they increased their autonomy. Additionally the authors note that there may be a positive impact on treatment adherence, although they acknowledge the need for a more stringent evaluation of all of these effects [36]. A final explanation for the null results of previous RCTs might be that the patient-held records did not consist of the necessary elements needed to improve patient outcomes. A paper version of the PHR was investigated in Stafford and Laugharne's [37] study, in which a patient-held care booklet was provided to long-term mental health service users including information on medications, contact numbers and notes. They found that the vast majority of those in the study viewed the booklet as useful and informative, with participants frequently citing the most useful information as telephone numbers and medication. Both of these were omitted from Warner et al.'s intervention [33]. Perhaps the most encouraging aspect of Stafford and Laugharne's design [37] is that a follow-up study five years later demonstrated that nearly 65% of those interviewed were still using the shared care record, showing naturalistic sustainability [38].

The potential benefits of ePHRs give good reason to pursue their design and implementation. None of the obstacles mentioned above such as computer literacy or issues of confidence are insurmountable; rather, they serve to illustrate that there are a number of issues which must be addressed before implementing ePHRs in the field of mental health. Little experimental rigour has been applied when investigating whether ePHRs will be acceptable and useful to both patient and clinician. There are a small number of naturalistic studies driving the implementation of ePHRs in mental health and although they are encouraging, the results must be viewed as anecdotal.

### An agenda for implementation

#### (i) Assessing technology

If a successful ePHR is to be introduced we must first be clear on the extent that service users have the ability to use the technology involved, both in terms of their computer literacy and their ability to access the technology. Irrespective of the trends in the general population towards greater understanding and use of technology we cannot assume that these trends are followed in the mental health service user population [18].

#### (ii) Including stakeholders in the system design

We need to know what it is that the stakeholder would like to see included in an ePHR, as well as how users are likely to interact with the resulting technological systems. The failure of HealthSpace is informative as it shows that policy-makers alone do not have the ability to predict what systems might be useful [14,15]. What might be valued by service users is exemplified in studies of the successful implementation of patient held records such as that of Stafford and Laugharne [37]. It is recommended that a user-centred design approach is taken, thereby ensuring that advisory groups consisting of the end users of such systems are centrally involved in all stages of system design and implementation following the guidance suggested by Trivedi and Wykes [39]. This will make their involvement meaningful, not merely for "consultation". Models for participatory research have also been developed in mental health and these are described in Rose, Evans, Sweeney and Wykes [40]. A number of the systems mentioned in the studies have also been hampered by a lack of health professional involvement. Any system must also be acceptable to health care staff if maximum efficacy is to be achieved. The health record is a partnership of care information and both parties must understand the benefits and have confidence in it. This is especially important given that the aim of e-health is often cited as empowering the patient and equalising their status with the practitioner [41], and this is unachievable if the ePHR is simply tokenism.

Recent work with in the UK with service users on what they would like from continuity of care [42] and similarly in the sorts of outcome information they would value [43] would also be of use as a starting point for ePHR designers.

#### (iii) Taking note of challenges

A major theme of the relevant literature is that receptivity to an ePHR is not necessarily homogenous across mental health service users. As a result care should be taken through piloting procedures to identify which sub-groups would most likely benefit from an ePHR. Although the current literature is scarce, the studies reported here suggest that some service user characteristics such as severe paranoia, poor introspection and older or less well-educated individuals might need additional support in using any such system. There may be other characteristics not yet discovered that require a slightly different approach. Likewise, it is important to understand and respect the choices and decisions of those professing a lack of interest and enthusiasm regarding ePHRs. Patient distress or upset appears a common occurrence when granting patients access to health information. This is a factor not only for service users, whose upset should be minimised as much as possible, but for health care professionals, who may feel torn between pressures for transparency and a responsibility for patient protection. The consequence may be that clinicians omit important information from their notes, resulting in less efficient shared care. Clear guidelines should therefore be in place to provide practitioners with the confidence that they can complete full written assessments without fear of culpability should the notes cause upset. It is clear that changes are needed at both the professional and organisational level in order to implement ePHRs. More research is needed on how these changes can best be supported. Confidence also depends on accountability. There must be clear instruction as to the influence of patient-reported data so that the user feels empowered, not encumbered.

#### (iv) Understanding ePHRs as part of dynamic systems

The evaluation of HealthSpace powerfully demonstrated what can happen if those planning and implementing ePHRs regard them 'statically' - i.e. see them simply as 'containers for data' [15]. The success or failure of any technological intervention is to a large part determined not by any putative inherent feature of the technology, but the manner in which it is put into circulation amidst existing networks and complex social relations. If ePHRs are to be successfully introduced to mental health care, those implementing them need to consider:

• service users' and healthcare professionals' existing use of and attitudes towards technology

• specific clinical, technological, organisational and financial contexts that will shape uptake of ePHRs

• how best to ensure that stakeholders are active contributors to - rather than passive recipients of - the planned technological innovations.

### The iterative process

The focus of these pioneering technological initiatives should not be restricted to their developmental phases. There is a need for continuous and rigorous assessment of e-health interventions whose potential will only be realised if implementation follows a logical progression with constant feedback [44] and ongoing, meaningful involvement of all stakeholders. Sittig and Classen warn of the dangers of the "aggressive" strategy of implementing electronic health records in the US, and call for a much more stringent assessment before they are introduced in order to ensure the safety and efficacy of such a system [45]. In fact, Sittig and Classen [45] suggest that a specific organisation is necessary to deal with the teething problems of electronic health records so that a high quality of care is maintained throughout their introduction.

An important part of assessing whether ePHRs are a success will be establishing which outcome measures should be chosen to monitor their utility. The differences between the naturalistic studies and the randomised trials could be resolved if there were at least the inclusion of some standard measures of what one would expect to change as a result of using an ePHR. Often the literature assumes that patient satisfaction, empowerment, and health status will improve as result of increased patient participation, although this is not always the case [46]. Instead it is more common that communication and condition management improve [46]. However, improved communication can be seen as a condition of empowerment if it hands over some choice and control to the service user. A wider approach to outcomes when assessing ePHRs may enable us to distinguish important moderating effects on outcomes such as differences between diagnostic groups, ages and levels of technological skill; in clinician-patient relationships; and in how ePHRs are embedded within specific care pathways and practices. The exploratory analyses of benefit will then allow further iterations to improve the implementation process. It is also vital to measure costs as well as benefits as these will lead to differing conclusion on cost utility, cost-benefit and cost-effectiveness. These will then provide a realistic view of the worth of such records.

### Future research

#### (i) What might be the benefits?

One benefit that is often cited is empowerment and this has frequently been the focus of the literature on personal health records, in general as well as in psychiatric care [47-50]. Unfortunately most reports assume that personal health records will implicitly 'empower' the patient, with no attempt to define exactly what is meant by the term, nor to consider that other outcomes might be possible (for example Greenhalgh et al. [15] argued that HealthSpace did not lead to greater empowerment, not least because the use of email-style messaging appeared to erode rather than enhance autonomy in some patients). In the ePHR literature 'empowerment' is assumed to be self-explanatory but there are a variety of definitions ranging from succinct, truncated versions such as 'autonomy' [36], 'self-efficacy' [51] or 'choice' [41] to lengthy explanations of the intra-personal, inter-personal and culture-wide *process *of empowerment. These represent two broad ideologies: those who believe that empowerment is something that can be conferred upon another, and those who believe that it is something that must come from within the individual. Undoubtedly self efficacy, choice and increased control are necessary but not sufficient conditions for empowerment, but in studies using these types of definition it is assumed that each individual factor is equivalent to empowerment. The lack of clarity on how ePHRs are supposed to increase empowerment and the setting conditions for this mean that future studies need to be much more explicit about the model they are adopting if empowerment is their key aim and there are models in other literatures that could be copied [40].

#### (ii) The potential financial and other costs

There are assumptions that ePHRs are always beneficial, and although we have already pointed out that there are risks in terms of the service user, we would like to see investigation of other potential costs. For instance, it is not entirely clear that the investment in ePHRs will bring monetary rewards such as reduced service costs that have been found in general medical conditions. In fact one study in mental health care did not find such reductions [33]. It may be that the levels of support required for some people with mental health problems to interact with this technology will outweigh any potential benefits that may accrue from less frequent visits. Equally medical staff and other professionals may need to be trained to make use of the ePHR, as well as needing to be informed of how to write in a patient-friendly way, adding more costs. Sidorov [52] argues that many of the forecasted savings are false, as decreases in the number of doctor visits are simply offset by decreases in productivity due to increased documentation. Sidorov also suggests that for the monetary savings to be realised, large numbers of staff would have to be laid off [52] leaving fewer staff to have the face to face contact valued by service users.

#### (iii) Conducting the research

The extant literature on personal health records, whilst sparse, contains widely heterogeneous methods making it difficult to delineate what makes some studies successful and others not. A recent review of the wider field of electronic patient records [53] shows how complicated the literature can become if researchers do not agree on comparable methods to assess efficacy. The same review emphasised the need for a collaborative approach, as multiple and varying domains will inevitably be involved in their design and implementation, posing questions of clinical utility, ethics and economics amongst others [53], and this is also our conclusion for the specific field of personal health records in mental health.

## Conclusions

The aim of this review is not to deter potential designers from pursuing ePHRs in mental health, nor is it to show that ePHRs are inappropriate for the population of mental health service users. It is intended to act as a reference point, and to demonstrate that a hasty implementation may well lead to costly results including a loss of confidence in ePHRs which may interfere with future access to technological innovation.

The ePHR may provide potential benefits for those who receive care from a range of services, as is frequently the case in mental health. The ePHR offers the opportunity of sharing information between services and with the individual service user, so that the information does not get lost and so that service users can play a greater role in the planning, recording and monitoring of their clinical care. The success of the Kaiser Permanente model and the failure of HealthSpace represent the poles of ePHR implementation, and research teams, designers and policy makers should learn from both before rushing into a wholesale, untailored approach to record sharing.

## Abbreviations

ePHR: electronic Personal Health Record; NHS: National Health Service; UK: United Kingdom; NICE: National Institute for Clinical Excellence; CBT: Cognitive Behavioural Therapy; MRC: Medical Research Council; NIHR: National Institute for Health Research; CRIS: Case Records Information System; US: United States of America; IT: Information Technology; GP: General Practitioner; DoH: Department of Health; RCT: Randomised Controlled Trial.

## Competing interests

The authors declare that they have no competing interests.

## Authors' contributions

LE researched and drafted the manuscript. TW conceived of the review. All authors helped to re-draft the article, read and approved the final manuscript.

## Pre-publication history

The pre-publication history for this paper can be accessed here:

http://www.biomedcentral.com/1471-244X/11/117/prepub
